# Targeting LDL Across the Whole Spectrum of Chronic Kidney Disease: From Pathophysiology to Novel Treatments

**DOI:** 10.1007/s11883-026-01434-y

**Published:** 2026-07-04

**Authors:** Roberto Minutolo, Luca De Nicola, Arturo Cesaro, Mario Cozzolino, Luca Di Lullo, Massimo Iacoviello, Ernesto Paoletti, Maura Ravera, Vincenzo Russo

**Affiliations:** 1 Division of Nephrology, Department of Advanced Medical and Surgical Sciences, University of Campania “Luigi Vanvitelli”, Naples, Italy; 2 Department of Translational Medical Sciences, University of Campania “Luigi Vanvitelli”, Naples, Italy; 3 Division of Cardiology, A.O.R.N. “Sant’Anna e San Sebastiano”, Caserta, Italy; 4 Renal Division, Department of Health Sciences, University of Milan, ASST Santi Paolo e Carlo, Milan, Italy; 5 Nephrology and Dialysis Unit, Azienda USL Roma 6, Albano Laziale, Italy; 6 Department of Medical and Surgical Science, University of Foggia, Foggia, Italy; 7 Nephrology and Dialysis Unit, Ospedale di Imperia, Imperia, Italy; 8 Clinica Nefrologica, Dialisi e Trapianto IRCCS Policlinico San Martino, Genoa, Italy; 9 Cardiology Unit, Monaldi Hospital, Naples, Italy; 10 Department of Translational Medical Sciences, University of Campania “Luigi Vanvitelli”, Naples, Italy

**Keywords:** Chronic kidney disease, Dyslipidemia, Cardiovascular risk, Statin, PCSK9 inhibitors

## Abstract

**Purpose of Review:**

The natural fate of chronic kidney disease (CKD) is the progression to dialysis; however, most patients face fatal and nonfatal cardiovascular events throughout their lifetime. We here address the role of low-density lipoprotein cholesterol (LDL-C) in the excess cardiovascular risk and the impact of traditional and innovative LDL-lowering therapies across the whole spectrum of CKD.

**Recent Findings:**

Current guidelines on the prevention of atherosclerotic cardiovascular disease (ASCVD) from European and US cardiology societies recommend the assessment of total cardiovascular disease risk to modulate the intensity of preventive strategies in relation to the cardiovascular risk of patients: the higher the cardiovascular risk, the more intense should be the intervention. New drugs have demonstrated efficacy in achieving the lower LDL-C goals not attained by traditional therapy. The detection of vulnerable coronary plaque, rather than merely be the presence of luminal narrowing, provides an attractive imaging target to guide intensified preventive strategies in high-risk population including patients with CKD.

**Summary:**

In the context of CKD, the patient journey represents a dynamic, longitudinal care pathway in which cardiovascular risk progressively increases in parallel with declining renal function. Despite clear recommendations from nephrology and non-nephrology guidelines, treatment initiation, maintenance and intensification as well as LDL-C target are frequently overlooked in CKD population. Since novel LDL-lowering therapies provide additional therapeutic options for the patients with CKD, it is today mandatory to raise awareness on cardiovascular risk and to integrate lipid management into the broader, longitudinal care of patients with CKD across all stages of disease.

## Introduction

Chronic Kidney Disease (CKD) is now recognized as global health priority: in May 2025, the World Health Organization (WHO) officially recognized the growing burden of CKD by adopting a landmark resolution to prioritize kidney health into national health strategies to target its rising prevalence and mortality. After few months, in September 2025, a political declaration tabled at the 80th United Nations General Assembly made the commitment to tackling CKD alongside other non-communicable disease (NCD). CKD has in fact overcome the other NCDs in terms of prevalence (10–14% in general population with about 850 million of patients worldwide), need of hospitalization, mortality rates, and costs for the National Health Services (40–50,000 euros/patient/year for treated end-stage kidney disease -ESKD) as well [1, 2]. While the natural fate of CKD is the progression to ESKD, most patients face fatal and nonfatal cardiovascular events throughout their lifetime. We here address the role of low-density lipoprotein cholesterol (LDL-C) in the excess CV risk and the impact of traditional and innovative LDL-lowering therapies across the whole spectrum of CKD.

## Cardiovascular Risk in CKD

**Fig. 1:**
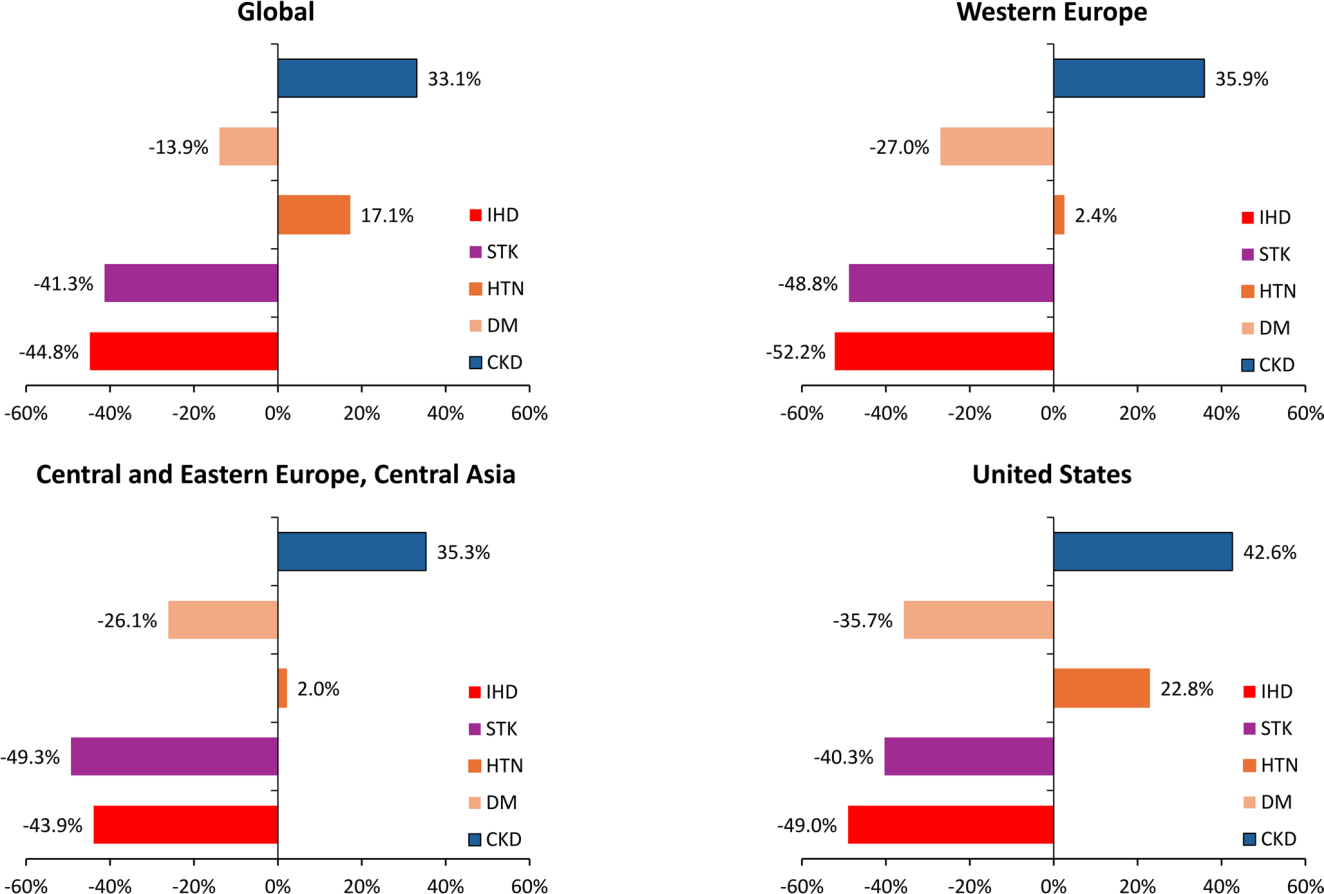
Projected mean percentage change from 2022 to 2050 in cause-specific age-standardized death rate, worldwide and by main geographical area [3] IHD, ischemic heart disease; STK, stroke; HTN, hypertension; DM, diabetes mellitus; CKD, chronic kidney disease

CKD today represents the tenth leading cause of death worldwide with a projected climbing at the fifth place by 2050 [3]. More importantly, the consistent growth of CKD-specific death rates contrasts with the decline of mortality caused by coronary heart disease (CHD), stroke, diabetes and the lower increase of hypertension-related death (Fig. 1) [3]. Therefore, CKD emerges as a unique NCD for which specific treatment strategies are today urgently needed.

Mortality in CKD is mainly driven by the high risk of both atherosclerotic and non-atherosclerotic cardiovascular disease, and high fatality after event as well, that accompanies patients throughout their entire disease journey, from the early CKD stages to treated ESKD [4–6]. This link is so strong that seminal studies identified CKD as a coronary artery disease (CHD) risk equivalent similar to diabetes [7, 8]. A large meta-analysis has now highlighted the strength of this association by disclosing a two- to four-fold higher multi-adjusted risk for cardiovascular mortality, CHD, stroke and peripheral artery disease in patients with moderate CKD (eGFR 59 − 45 mL/min/1.73m2 and urinary albumin to creatinine ratio -UACR 10–29 mg/g) [9]. This finding becomes more important when considering that the earlier stages of CKD are becoming more prevalent due to the implementation of the novel nephroprotective drugs that can remarkably slow CKD progression to treated ESKD [10]. Therefore, it is expected that the implementation of LDL-lowering therapies will become more frequent in the large population of CKD patients at early stages of disease.

Risk factors leading to the high cardiovascular risk in CKD span from traditional risk factors, including dyslipidemia, to various nontraditional risk factors, including oxidative stress, inflammation with or without protein-energy wasting, mineral disorders, and endothelial dysfunction whose importance increases with worsening kidney function [11]. Knowledge of the prognostic role of LDL-C is essential to facilitate clinical decision-making. However, interpretation in the CKD population can be problematic due to the multifactorial nature of progressive cardiovascular damage that limits the predictive value of a single traditional risk factor [12], as well as to the confounding effect of inflammation and malnutrition whose prevalence increases with worsening renal function, which is per se associates with lower LDL-C levels [13]. On the other hand, a linear correlation between cardiovascular risk and LDL-C level becomes manifest in renal clinics dedicated to management of non-dialysis CKD patients where multifaceted therapy is regularly implemented to prevent all complications including malnutrition [14].

## Risk Stratification in Patients With CKD

Current guidelines on the prevention of atherosclerotic cardiovascular disease (ASCVD) from European and US cardiology societies recommend the assessment of total cardiovascular risk to modulate the intensity of preventive strategies in relation to the cardiovascular risk of patients: the higher the cardiovascular risk, the more intense should be the intervention [15, 16].

As first step, primary prevention of ASCVD (including previous myocardial infarction or angina, revascularization procedures, stroke and TIA, and peripheral arterial disease) requires the evaluation of the risk profile of patients. Risk stratification is mainly based on the assessment of the individual risk prediction. The 2025 Update of European Society of Cardiology (ESC) and the European Atherosclerosis Society (EAS) Guidelines introduced two novel prediction models, one for patients aged 40–69 years (Systematic COronary Risk Evaluation 2, SCORE2) and one for patients aged ≥ 70 years (SCORE2-Older Persons, SCORE2-OP) to estimate the 10‑year risk of fatal or non‑fatal atherosclerotic events [17]. These models replace the older tool (SCORE1) that included only fatal cardiovascular events, while contemporary epidemiological picture in Europe has shifted towards a majority of non-fatal events. In U.S., the new recommended risk estimation model is the Predicting Risk of cardiovascular disease EVENTs (PREVENT) Equations [16], and, accordingly, patients are classified in four cardiovascular risk categories, low, borderline, intermediate and high risk according to US Guidelines and low, moderate, high and very-high risk according to ESC/EAS guidelines, with different thresholds (Table 1). SCORE2/SCORE2‑OP should not be applied to those patients already on lipid therapy or with established ASCVD/CKD; nonetheless, by recalibrating population risk, SCORE2 may change thresholds for treatment decisions near decision boundaries. Notably, the use of SCORE2-SCORE2-OP is also not recommended when CKD is present in patients without known ASCVD, as CKD is a major driver of ASCVD. In addition to the use of prediction model, several clinical condition may help risk stratification. On this regard, CKD has different weight in stratifying cardiovascular risk between ESC/EAS and American College of Cardiology (ACC) and American Heart Association (AHA) guidelines. Indeed, despite ACC/AHA Task Force considers patients with eGFR < 60 mL/min/1.73m2 (CKD stage 3 or higher) as those having established ASCVD, they recommend only moderate-intensity statin therapy with or without ezetimibe. A more aggressive approach (high-intensity statin therapy ± ezetimibe and/or PCSK9 inhibitors) is advised only in patients with CKD stage 3 or higher and clinical ASCVD [16]. In patients with diabetes, the presence of either eGFR < 60 mL/min/1.73m2 or albuminuria > 30 mg/g is considered a risk-enhancing factor independent of other diabetes-related risk factors [16]. Conversely, ESC/EAS guidelines deemed either moderate (eGFR 30–59 mL/min/1.73 m2) or severe CKD (eGFR < 30 mL/min/1.73 m2) as conditions that per se identify patients as high and very-high risk, respectively. Unfortunately, both guidelines did not include albuminuria as specific marker of cardiovascular risk for non-diabetic patients [16, 17]. The presence of a high and very high risk is also based on the coexistence of the following criteria: clinical ASCVD or on imaging ASCVD (significant plaque on coronary angiography or CT scan or on carotid ultrasound), diabetes mellitus, the presence of other major risk factors, high blood pressure levels, familial hypercholesterolemia. Finally, several risk modifiers have been proposed to refine classification, such as coronary artery calcium (CAC) score, family history of premature CVD, persistently elevated high-sensitive C-reactive protein (hs‑CRP), and elevated Lipoprotein a (Lp(a)). As shown in Table 1, risk stratification is the cornerstone for defining the level of LDL‑C requiring pharmacological treatment, as well as the therapeutic targets. In the latest version of ESC guidelines, pharmacological treatment must be initiated in all patients with very-high risk for secondary prevention and in those with LDL-C > 70 mg/dL in primary prevention [17]. High-risk patients with LDL-C > 100 mg/dL are also candidates to therapy. LDL-C target progressively decrease as cardiovascular risk profile increase and it contains a combination of LDL-C level (< 55 or 70 mg/dL in very-high and high risk, respectively) coupled with a percentage decline ≥ 50% from baseline [15, 17]. To achieve these targets, the 2025 update explicitly incorporates newer agents. Specifically, while reiterating statins as first line, it emphasizes the need of adding non‑statin therapies with proven CV benefit when goals are unmet, with the choice based on the magnitude of LDL‑C lowering desired. Moreover, in acute coronary syndromes, early intensive LDL‑C lowering to achieve goals sooner (“*strike early and strong*”) is advised [17]. US guidelines, recommend similar LDL-C goal in high-risk patients [16].

**Table 1:** Guideline recommendations for risk stratification, start of therapy and target of LDL-C

	Risk categories	Start of drug therapy	LDL-C target (mg/dL)
ESC/EAS 2025	• Extreme: Patients with ASCVD who experience recurrent vascular events despite statin-based therapy or patients with polyvascular arterial disease	• Intensify statin-based therapy	• < 40 & ≥50% decline
	• Very high: SCORE2/SCORE2-OP > 20%, ASCVD, eGFR < 30 mL/min/1.73 m^2^, T2DM with microalbuminuria, retinopathy, or neuropathy, early onset of T1DM lasting > 20 years	• Recommended if LDL-C > 70 mg/dL;	• < 55 & ≥50% decline
	• High: SCORE2/SCORE2-OP 10%-20%, DM without TOD lasting ≥ 10 years or another additional risk factor, eGFR 30–59 mL/min/1.73 m^2^, markedly elevated single risk factors, (LDL-C > 190 mg/dL, or BP ≥ 180/110 mmHg)	• Considered if LDL-C 55–70 mg/dL	• < 70 & ≥50% decline
	• Moderate SCORE2/SCORE2-OP 2%-9%, young patients (T1DM < 35 years; T2DM < 50 years) with DM duration < 10 years, without other risk factors	• Recommended if LDL-C > 100 mg/dL	• < 100
	• Low: SCORE2/SCORE2-OP < 2%	• Consider if LDL-C 70–100 mg/dL	• < 116
		• Consider if LDL-C 100–190 mg/dL	
		• Consider if LDL-C 100–190 mg/dL	
ACC/AHA 2026	Secondary prevention		
	• Adults at very high risk (multiple major ASCVD events or 1 major ASCVD event + ≥ 2 high-risk conditions)*, Adults with CKD Stage ≥ 3 and ASCVD	• High intensity statin±ezetemibe and/or PCSK9i	• < 55 & ≥50% decline
	• Adults at high risk and those with CKD Stage ≥ 3 without ASCVD	• High intensity statin (add ezetimibe, PCSK9i or bempedoic acid if LDL-C goal not met)	• < 70 & ≥50% decline
	Primary prevention (10-y risk estimate based on PREVENT-ASCVD equation)		
	• High: 10-y risk of ASCVD ≥ 10%, LDL-C > 190 mg/dL, T2DM	• Recommend high intensity statin±ezetemibe	• ≥ 50% decline
	• Intermediate: 10-y risk of ASCVD 5%-<10%).	• Consider moderate intensity statin**	• 30–49% decline
	• Borderline: 10-y risk of ASCVD 3%-<5%	• Consider moderate intensity statin**	• < 100 & 30–49% decline
	• Low: 10-y risk of ASCVD < 3%	• Moderate intensity statin (if LDL-C 160–189)	
KDIGO 2024	• Age > 50 years and eGFR < 60 mL/min/1.73 m^2^	• Recommend statin±ezetemibe	• No LDL-C target recommended (*fire and forget* strategy)
	• Age > 50 years and eGFR < 60 mL/min/1.73 m^2^	• Consider statin	
	• Age ≤ 50 years, the risk profile for starting treatment consider also the presence of ASCVD, T2DM and estimated 10-year incidence of cardiovascular event > 10% (by using predictive models developed in CKD population or that include eGFR and Albumin-Creatinine Ratio)	• Recommend statin±ezetemibe	

* High-risk conditions: age>65y, history of coronary artery bypass graft; PCI percutaneous coronary intervention, current smoker, diabetes, congestive heart failure, high blood pressure (BP), LDL ≥ 100 mg/dL. ** Main risk enhancer factors are: premature ASCVD in a parent or sibling, chronic inflammatory diseases, Lp(a) ≥ 50 mg/dL, hsCRP ≥ 2 mg/L on > 1 occasion, triglycerides persistently ≥ 175 mg/dL (or ≥ 150 mg/dL if fasting), CKM syndrome, LDL-C persistently ≥ 160–189 mg/dL. Their presence may help to stratify risk thus supporting a decision to initiate or intensify statin therapy. Abbreviations: ASCVD, atherosclerotic cardiovascular disease; SCORE2, Systematic COronary Risk Evaluation 2; SCORE2-OP, SCORE2-Older Persons; DM, diabetes mellitus; T2DM, type 2 diabetes mellitus; T1DM, type 1 diabetes mellitus; TOD, target organ damage; PREVENT, Predicting Risk of cardiovascular disease EVENTs

Guidelines specifically dedicated to nephrologists (KDIGO 2024) [18] recommend the use of new models specifically developed in CKD population (QRISK3 and ckdpc.org) or modified existing risk-prediction models that include eGFR and albuminuria to predict cardiovascular events in individuals with CKD [19–21]. However, for recommending start of treatment, a greater importance seems to be attributed to kidney measures since KDIGO 2024 recommend the use of statin with or without ezetimibe in patients older than 50 years and eGFR < 60 (Grade 1 A) or > 60 mL/min/1.73 m2 (Grade 1B) without considering albuminuria level [18]. For younger patients, the risk profile to decide start of treatment also considers the presence of coronary or cerebrovascular events, diabetes and estimated 10-year incidence of cardiovascular event > 10% (Grade 2 A). Major difference with cardiology guidelines relates to the goal of LDL-C to reach with treatment. On this regard, KDIGO 2024 maintains the indications of KDIGO guidelines on dyslipidemia management issued in 2013, which were based on the “*fire and forget”* approach without suggesting either a specific goal of LDL-C or a specific timing of measurement [22]. Conversely, ESC/EAS Guidelines recommend measuring LDL-C 4–6 weeks after initiation or intensification of lipid-lowering therapy, then periodically to assess the LDL‑C response. Moreover, adherence, attention to drug-drug interactions, and individualized selection of adjunctive non‑statin agents are central to implementing the recommendations.

## Lipid Profile in Patients With CKD

Dyslipidemia represents a common metabolic complication of CKD largely contributing to the high cardiovascular risk in this population. Unlike the general population, lipid abnormalities in CKD are predominantly qualitative and functional, rather than quantitative, thus reflecting profound disturbances in lipoprotein metabolism [23].

In early and intermediate stages of non-dialysis CKD, typical lipid profile is characterized by hypertriglyceridemia, storage of triglyceride-rich lipoproteins -particularly very-low-density lipoproteins (VLDL) and remnant particles- reduced level of high-density lipoprotein cholesterol (HDL-C), together with qualitative imbalance in LDL particles. Although LDL-C levels are often normal or mildly elevated, LDL particles are predominantly small and dense, more susceptible to oxidation and glycation, and highly atherogenic [24]. This pattern contributes to the so-called “lipid paradox” (Table 2), whereby apparently normal LDL-C concentrations are associated with the well-known markedly increased cardiovascular risk [25].

**Table 2:** Lipid profile in patients with chronic kidney disease (CKD)

Lipid Parameter	Characteristic in CKD	CV Impact	Prevalence in CKD
Total Cholesterol	Normal or mildly reduced	Moderate	65–70%
LDL-Cholesterol	Normal or reduced*	Paradoxical*	37–40%
HDL-Cholesterol	↓ Reduced	Very High	90%
Triglycerides	↑ Elevated	Very High	86–90%
VLDL-Cholesterol	↑ Elevated	High	Relevant
Apolipoprotein B	↑ Elevated	Very High	Increased
Apolipoprotein A1	↓ Reduced	Very High	Reduced
Lipoprotein(a)	↑ Accumulated (reduced clearance)	Very High	Accumulated
Small Dense LDL	↑ Increased atherogenic particles	Very High	Prevalent

*Lipid Paradox: Normal/Low LDL-Cholesterol but elevated cardiovascular risk

The alteration in HDL-C is not limited to quantitative reduction; it involves deep functional impairment, including impaired antioxidant and anti-inflammatory properties and diminished capacity of cholesterol efflux. These abnormalities are driven by chronic inflammation, oxidative stress, and changes in apolipoprotein composition. Proteinuria further amplifies the dyslipidemia by stimulating hepatic lipoprotein synthesis, thereby establishing a bidirectional relationship between renal dysfunction and lipid metabolism [26].

As CKD progresses to ESKD, lipid imbalance become more pronounced and qualitatively distinct [27]. Individuals treated by hemodialysis or peritoneal dialysis commonly exhibit severe hypertriglyceridemia, marked accumulation of remnant lipoproteins, persistent elevation of lipoprotein(a) due to impaired renal clearance, and profound HDL-C dysfunction, with particles that may acquire pro-atherogenic properties. In advanced CKD, LDL-C levels often decline, as a consequence of malnutrition-inflammation syndrome, thereby limiting reliability of this traditional biomarker for cardiovascular risk stratification [28].

Peritoneal dialysis further contributes to the metabolic derangements because chronic glucose absorption from the dialysate enhances hepatic lipogenesis therefore exacerbating hypertriglyceridemia [29].

Overall, prevalence of dyslipidemia may reach up to 90% of patients with severe CKD. The underlying pathophysiological mechanisms include insulin resistance, reduced lipoprotein lipase activity, accumulation of uremic toxins, systemic inflammation, and oxidative stress. These features explain why conventional lipid metrics incompletely predict cardiovascular risk in advanced CKD and point up to the need for stage-specific interpretation of lipid profile, tailored therapeutic strategies and early monitoring -including apolipoproteins to lipid ratio- in order to better predict cardiovascular outcome in this high-risk population [30].

## Treatment of Dyslipidemia in Patients With Chronic Kidney Disease (CKD)

A broad therapeutic armamentarium with proven cardiovascular benefit is currently available, offering different mechanisms of action and various degrees of efficacy in reducing LDL-C. Moreover, combining different drugs results in a synergistic effect that can lower LDL-C more effectively and rapidly than high-dose monotherapy alone (Table 3).

**Table 3:** Mechanism of action and reduction in LDL-C levels with different class of drugs

Drug Class	Mechanism of Action	Primary target	LDL-C Reduction	Route/Frequency
Statins	HMG-CoA Reductase inhibition	Hepatic synthesis		Oral/daily
• Low intensity ^1^			< 30%	
• Moderate Intensity ^2^			30–49%	
• High Intensity ^3^			≥ 50%	
Ezetimibe	NPC1L1 Inhibition	Intestinal absorption	∼ 20% *	Oral/daily
Bempedoic Acid	ATP-Citrate Lyase Inhibition	Hepatic synthesis (Upstream of HMG-CoA reductase)	∼ 23%	Oral/daily
PCSK9 mAbs	PCSK9 Neutralization	LDL-R Recycling	∼ 60%	S.C./Every 2–4 weeks
Enlicitide	PCSK9 Neutralization	LDL-R Recycling	∼ 60%	Oral/daily
Inclisiran	siRNA-mediated PCSK9 gene silencing	LDL-R Recycling	∼ 50%	S.C./second dose at 3 months, then every 6 months

Abbreviations: *HMG-CoA* 3-hydroxy-3-methylglutaryl-coenzyme A, *NPC1L1* Niemann-Pick C1 like 1 protein, *PCSK9 mAbs* proprotein convertase subtilisin/kexin type 9 monoclonal antibodies, *LDL-R* receptor of LDL-C, *S.C.* subcutaneous. * in combination with High Intensity Statins LDL-C reduction is ∼60%

Definition of intensity of statin treatment is reported according to [16]

^1^Low-Intensity Statin includes: Simvastatin 10 mg, Fluvastatin 20–40 mg, Pravastatin 10–20 mg, Lovastatin 20 mg;

^2^Moderate-Intensity Statin includes: Atorvastatin 10–20 mg, Rosuvastatin 5–10 mg, Simvastatin 20–40 mg, Pravastatin 40–80 mg, Lovastatin 40–80 mg, Fluvastatin XL 80 mg, Pitavastatin 1–4 mg

^3^High Intensity Statins: Atorvastatin 40–80 mg, Rosuvastatin 20–40 mg

### Evidence for LDL-Lowering Therapy in Non-Dialysis CKD

Statins are the cornerstone of lipid-lowering therapy, since they are effective in reducing cardiovascular and all-cause mortality and preventing major cardiovascular events in patients with CKD stage 1–4, with the greatest benefit observed in the initial stages of renal disease [31, 32]. The landmark Study of Heart And Renal Protection (SHARP) investigated the efficacy of statin plus ezetimibe in 9,270 patients with stage 3–5 CKD (6,247 not on dialysis; 3,023 on dialysis) and no history of CHD [33]. Subjects were randomized to receive simvastatin 20 mg plus ezetimibe 10 mg or placebo. Over a median follow-up of 4.9 years, simvastatin/ezetimibe compared to placebo reduced the risk of the primary composite endpoint (non-fatal myocardial infarction, coronary death, non-hemorrhagic stroke, or any arterial revascularization procedure) by 17%, with a good safety profile. Consistent with these findings, a sub-analysis of the IMPROVE-IT study in patients with ASCVD, LDL-C < 125 mg/dl and CKD confirmed a significant and even greater absolute benefit from the addition of ezetimibe to simvastatin as compared to simvastatin monotherapy [34].

Two large trials evaluated the effectiveness of Proprotein convertase subtilisin/kexin type 9 (PCSK9) inhibitors. The first study (Further Cardiovascular Outcomes Research with PCSK9 Inhibition in Subjects with Elevated Risk, FOURIER trial) evaluated 27,564 patients with a history of MI, stroke, or peripheral arterial disease and LDL-C ≥ 70 mg/dL randomized to receive either evolocumab (140 mg every 2 weeks or 420 mg monthly) or placebo, in addition to moderate-to-high intensity statin therapy [35]. Over a median follow-up of 2.2 years, patients receiving evolocumab showed a rapid and sustained LDL-C reduction throughout follow-up (-59% at week-48) and, more importantly, a 15% risk reduction in the primary composite endpoint, of cardiovascular death, myocardial infarction (MI), stroke, hospitalization for unstable angina, or coronary revascularization [35].

Similar findings were observed with alirocumab compared to placebo in the ODYSSEY OUTCOMES trial [36]. Over a median follow-up of 2.8 years, alirocumab induced a 55% LDL-C reduction at week-48 and significantly reduced by 15% the risk of a composite cardiovascular outcome. The benefit was higher in patients with higher baseline serum cholesterol or in those at very high cardiovascular risk [36, 37]. In both trials, patients with advanced CKD were excluded (i.e., those with eGFR < 20 and < 30 mL/min/1.73m^2^ in FOURIER and ODISSEY, respectively). However, in post-hoc analysis of the FOURIER trial, LDL-C lowering and cardiovascular protection of evolocumab versus placebo were consistently significant across CKD subgroups (-18%, in patients with preserved renal function − 15% in CKD stage 2 and − 11%, in CKD stage 3–4, P for interaction 0.77) [38]. Despite alirocumab actually reduced LDL-C levels irrespective of baseline renal function [39], cardiovascular benefit observed in patients with normal renal function (HR 0.78, 95%CI 0.67–0.92; *P* = 0.003) and in those with CKD stage 2 (HR 0.83, 95%CI 0.73–0.95; *P* = 0.006) disappeared in patients with CKD stage 3 (HR 0.97, 95%CI 0.81–1.18; *P* = 0.784) [40].

Inclisiran is a first-in-class, long-acting small interfering RNA (siRNA) that reduces LDL-C by inhibiting the hepatic production of PCSK9 protein. In the ORION-7 trial and pooled analyses of the ORION program, inclisiran demonstrated a robust and consistent LDL-C reduction of approximately 50%, regardless of the degree of renal impairment [41, 42]. Moreover, as demonstrated for PCSK9 inhibitors, no dosage adjustment is required according to renal function. In fact, although plasma concentration increases proportionally with the severity of renal impairment, the drug is rapidly cleared from the circulation within 24–48 h due to its highly selective hepatic uptake [41]. However, the impact of inclisiran on major adverse cardiovascular events is currently unknown. Three ongoing large placebo-controlled randomized trials will assess the effects of inclisiran on clinical outcomes among people with either pre-existing ASCVD (NCT03705234 and NCT05030428) or in primary prevention (NCT05739383).

Bempedoic acid is a prodrug activated only in the liver that inhibits ATP citrate lyase, an enzyme upstream of HMG-CoA reductase (same target of statins) in the cholesterol synthesis pathway. Bempedoic acid lowers LDL-C levels similarly to statins by reducing hepatic cholesterol synthesis and raising LDL receptor expression [43]. In the Cholesterol Lowering via Bempedoic Acid [ECT1002], an ACL-Inhibiting Regimen (CLEAR Outcomes) trial, during a median follow-up of 40.6 months, bempedoic acid significantly lowered LDL-C by 26%, hs-CRP by 19% and the cardiovascular risk (composite of cardiovascular death, nonfatal myocardial infarction, nonfatal stroke, or coronary revascularization) by 13% in statin-intolerant patients. This CV benefit is maintained in patients with CKD stage 3 [44]. Of note, there is currently limited data for patients with eGFR < 30 mL/min/1.73 m². Furthermore, a reversible, modest increase in serum creatinine and a corresponding decrease in eGFR may be observed with bempedoic acid therapy, probably due to inhibition of creatinine tubular secretion [45].

### Evidence for LDL-Lowering Therapy in Dialysis CKD

Patients with ESKD treated by maintenance dialysis represent the highest-risk population for cardiovascular morbidity and mortality across the whole spectrum of CKD, with cardiovascular disease accounting for nearly 50% of deaths [46, 47]. However, LDL lowering remains particularly controversial in this setting for four reasons. First, in dialysis patients LDL-C levels are often normal or modestly reduced, while low HDL-C and hypertriglyceridemia -the latter especially in peritoneal dialysis- predominate [27, 29]. Second, LDL-C particles undergo qualitative modifications, including increased oxidation, glycation, and enrichment with small dense LDL-C sub fractions [48], which enhance atherogenicity and endothelial dysfunction. Third, lower LDL-C and total cholesterol levels in dialysis can reflect inflammation, malnutrition, and consequent protein-energy wasting [11, 49]. Fourth, sudden cardiac death, heart failure, arrhythmias, and vascular calcification contribute to adverse outcomes, thereby diluting the clinical impact of therapies that target LDL-C-mediated plaque progression alone [50].

Consequently, LDL-C may be a poor standalone marker of atherosclerotic cardiovascular risk in dialysis patients. Accordingly, the three historical randomized trials including dialysis patients have consistently demonstrated that statin therapy is safe and efficacious in attaining substantial reductions in LDL-C without demonstrating improvement in cardiovascular or all-cause mortality. The Die Deutsche Diabetes Dialyse Studie (4D) trial evaluated atorvastatin in patients with type 2 diabetes undergoing hemodialysis. Despite an approximately 40% reduction in LDL-C, atorvastatin did not reduce the primary composite outcome of cardiovascular death, nonfatal myocardial infarction, or stroke [51]. Similarly, the AURORA trial demonstrated that rosuvastatin substantially lowered by 43% LDL-C in hemodialysis patients but failed to reduce major cardiovascular events or all-cause mortality [52]. The SHARP trial assessed simvastatin plus ezetimibe in a broad CKD population. Again, while the overall cohort experienced a significant 30% reduction in major atherosclerotic events, subgroup analyses of patients receiving dialysis did not demonstrate any significant benefit [33].

Nevertheless, post-hoc analyses of 4D and AURORA produced interesting (and more soundness) findings. In 4D patients, LDL-C lowering provided significant cardiac protection in the presence of very high LDL-C (> 145 mg/dL); Authors concluded that the basal levels of LDL may likely condition the treatment effect [53]. Indeed, SHARP had evidenced that cardiovascular protection is significant only if LDL-C is high (≥ 116 mg/dL) [33]. Similarly, the post-hoc study by AURORA investigators showed that rosuvastatin significantly reduces the rates of cardiac events by 32% among patients with diabetes [54].

Collectively these analyses indicate that LDL-lowering therapy by statins with or without ezetimibe might reduce the risk of fatal and nonfatal cardiac events in dialysis patients characterized by a higher cardiovascular risk associated with high LDL-C level or diabetes. Data on newer agents, including bempedoic acid, PCSK9 inhibitors and inclisiran, are lacking in dialysis because these patients were excluded from trials and their clinical role remains speculative pending outcome-driven studies. Current evidence supports LDL-lowering therapy after dialysis onset based on a patient-centered approach with concomitant scrutiny of cardiovascular risk, including LDL-C levels and diabetic status, and nutritional status.

Recently, The Protection against Incidences of Serious Cardiovascular Events Study (PISCES) trial randomized 1,228 hemodialysis patients to receive daily supplementation with fish oil or corn oil placebo [55]. During 3.5 years of follow-up, the risk of serious cardiovascular events (composite of cardiovascular death, nonfatal and fatal myocardial infarction, nonfatal and fatal stroke, and peripheral vascular disease leading to amputation) was significantly reduced in the fish-oil group by 43% with similar protective effect on each component of primary outcome. Therefore, this treatment could be used to optimize further the cardiovascular protection in this high-risk population.

### Lipid-Lowering Therapy in Kidney Transplant Recipients (KTRs)

CHD greatly accounts for the excess of cardiovascular mortality observed in KTRs [56]. Among traditional factors, dyslipidemia plays a major role in the CHD of KTRs since immunosuppressive agents, especially glucocorticoids, cyclosporine, and mTOR inhibitors, are associated with abnormalities in lipid profile with increase in total cholesterol, and LDL-C, whereas tacrolimus may contributes by inducing post-transplant diabetes mellitus (PTDM). Furthermore, improvement in general conditions with increase in food consumption may further predispose to dyslipidemia.

Historical strategies aimed at improving lipid profile after kidney transplantation (KTx) include corticosteroid therapy minimization, either steroid-sparing or discontinuation in immunosuppressive protocols [57]. Positive effects on lipid profile, however, must be carefully balanced by the increased risk of graft rejection. Introduction of mTOR inhibitors to reduce the risk of malignancies and viral infection, namely CMV and BKV, should be considered with caution due to the high risk of hyperlipidemia. Last, belatacept with avoidance of CNIs can reduce the risk of PTDM and dyslipidemia though it increases the risk of rejection and lymphoproliferative disorders [58].

Efficacy of lipid-lowering therapy in KTRs has been poorly investigated. The ALERT Trial evaluated 2102 KTRs with elevated LDL-C who were randomly assigned in a 1:1 ratio to fluvastatin 40 mg/day or placebo [59]. Although a 32% reduction in LDL-C was observed in KTRs on active arm, no difference as compared to controls was reported in the primary endpoint (composite of cardiac death, non-fatal myocardial infarction or the occurrence of coronary revascularization). Nonetheless, fewer cardiac deaths and MI in the fluvastatin arm were detected. By contrast, in the 2-year open-label extension of the ALERT study, patients receiving fluvastatin had 21% reduced risk of MACE and 29% reduction in cardiac death or non-fatal MI [60], thus supporting the use of statins in KTRs. This strategy has been confirmed by a later meta-analysis of 22 studies including 3,465 participants [61].

Nevertheless, statins metabolism may interfere with metabolism of both CNIs and mTOR inhibitors, thus claiming for caution in their systematical adoption in KTRs. Although data on ezetimibe are scanty, effectiveness and safety of this drug (in monotherapy or associated to statins) have been reported [62].

KTRs should be considered high or very high-risk patients, in whom a more aggressive therapeutic approach based on association of ezetimibe, statins and PCSK9 inhibitors is recommended to prevent MACE [63]. On this regard, preliminary data in KTRs have shown that evolocumab effectively reduces LDL-C as well as the incidence of cardiovascular events [64]. Interestingly, no effects on both eGFR level and tacrolimus trough levels were observed with evolocumab, thus highlighting the opportunity of considering such a new strategy in KTRs at higher cardiovascular risk. Of course, well-designed and powered trials exploring hard cardiovascular outcome in KTRs are warranted aimed at confirming these promising preliminary findings in an effort to lower the impact of CHD on clinical outcome of KTRs.

### Atherosclerotic Plaque in CKD: Prognostic Significance and Therapeutic Approach

Atherosclerotic plaque detection has emerged as a pivotal tool to refine cardiovascular risk stratification in patients with CKD where traditional scores consistently underestimate risk [65]. Beyond the high prevalence of asymptomatic coronary and carotid disease in this population, imaging of subclinical atherosclerosis captures the cumulative burden of dyslipidemia, inflammation, and CKD‑related vascular damage, providing information that is complementary to standard clinical variables [66]. The CAC score and coronary computed tomography angiography (CCTA) are particularly relevant in this setting, as they allow the identification of non‑obstructive but biologically active coronary atherosclerosis that may not be apparent on functional testing [67, 68].

Although carotid ultrasound data have consistently shown that the presence and extent of plaque predict cardiovascular events in CKD, the most clinically meaningful information for decision-making increasingly comes from coronary imaging. CCTA and intravascular imaging techniques have demonstrated that patients with CKD frequently have a high burden of non-obstructive, lipid-rich plaques characterized by positive remodeling, low-attenuation core, spotty calcification, and thin fibrous caps [68–70]. This vulnerable plaque phenotype, superimposed on the background of medial calcification and arterial stiffness typical of CKD, helps explaining the excess of myocardial infarction and sudden cardiac death that is not fully captured by stenosis severity alone [71]. Therefore, the detection of vulnerable coronary plaque, rather than merely be the presence of luminal narrowing, provides an attractive imaging target to guide intensified preventive strategies in this very high-risk population.

From a therapeutic standpoint, the identification of coronary (or carotid) plaque in CKD should not be viewed as a purely diagnostic finding but as a trigger for the intensification of cardiovascular prevention. Current ESC/EAS dyslipidemia guidelines classify severe CKD as a very high-risk condition and consider documented atherosclerotic cardiovascular disease by imaging as equivalent to clinical events, supporting low-density lipoprotein cholesterol targets of < 55 mg/dL with at least a 50% reduction from baseline [17]. In patients with CKD and imaging evidence of extensive or vulnerable plaque, a stepwise strategy including high-intensity statins and ezetimibe, followed by the early introduction of PCSK9 inhibitors or siRNA when targets are not achieved, should therefore be prioritized. In dialysis and post-transplant settings, where randomized outcome data are more limited and vascular calcification is prominent, coronary imaging may still help at individualizing treatment by identifying those with a particularly high atherosclerotic burden who are most likely to benefit from aggressive lipid lowering, alongside meticulous control of blood pressure, mineral metabolism, and glycaemia [72].

Intravascular ultrasound and optical coherence tomography studies have shown that intensive LDL-C lowering with PCSK9-targeting agents reduces overall atheroma volume and increases fibrous cap thickness and decreases lipid burden, consistent with plaque stabilization [73]. A growing body of intravascular imaging studies has shown that intensive LDL-C lowering with PCSK9 inhibitors favorably modulates coronary plaques in addition to improving clinical outcomes. In the GLAGOV trial, evolocumab, in addition to high-intensity statin therapy, significantly reduced percent atheroma volume over 18 months, with two-thirds of treated patients showing overall plaque regression, whereas nearly half of statin-only patients failed to regress [74]. Subsequent trials, such as PACMAN-AMI and HUYGENS, using a combination of intravascular ultrasound, optical coherence tomography, and near-infrared spectroscopy, demonstrated that alirocumab or evolocumab added to rosuvastatin not only induced further plaque volume reduction but also increased fibrous cap thickness and decreased lipid burden, consistent with a more stable plaque phenotype [75, 76]. These data support the concept that PCSK9 inhibition can both shrink and stabilize high-risk coronary plaques, a mechanism that is particularly attractive in patients with CKD, in whom plaques are often extensive, inflamed, and heavily calcified.

Although dedicated imaging trials in CKD are limited, the extrapolation of these findings to this high-risk population is biologically plausible, given the very high risk associated with CKD and the high prevalence of subclinical, often vulnerable, coronary plaques in this setting [18]. In addition to oral-centered strategies, injectable therapies with complementary mechanisms may further contribute to plaque stabilization and risk reduction in CKD.

**Fig. 2:**
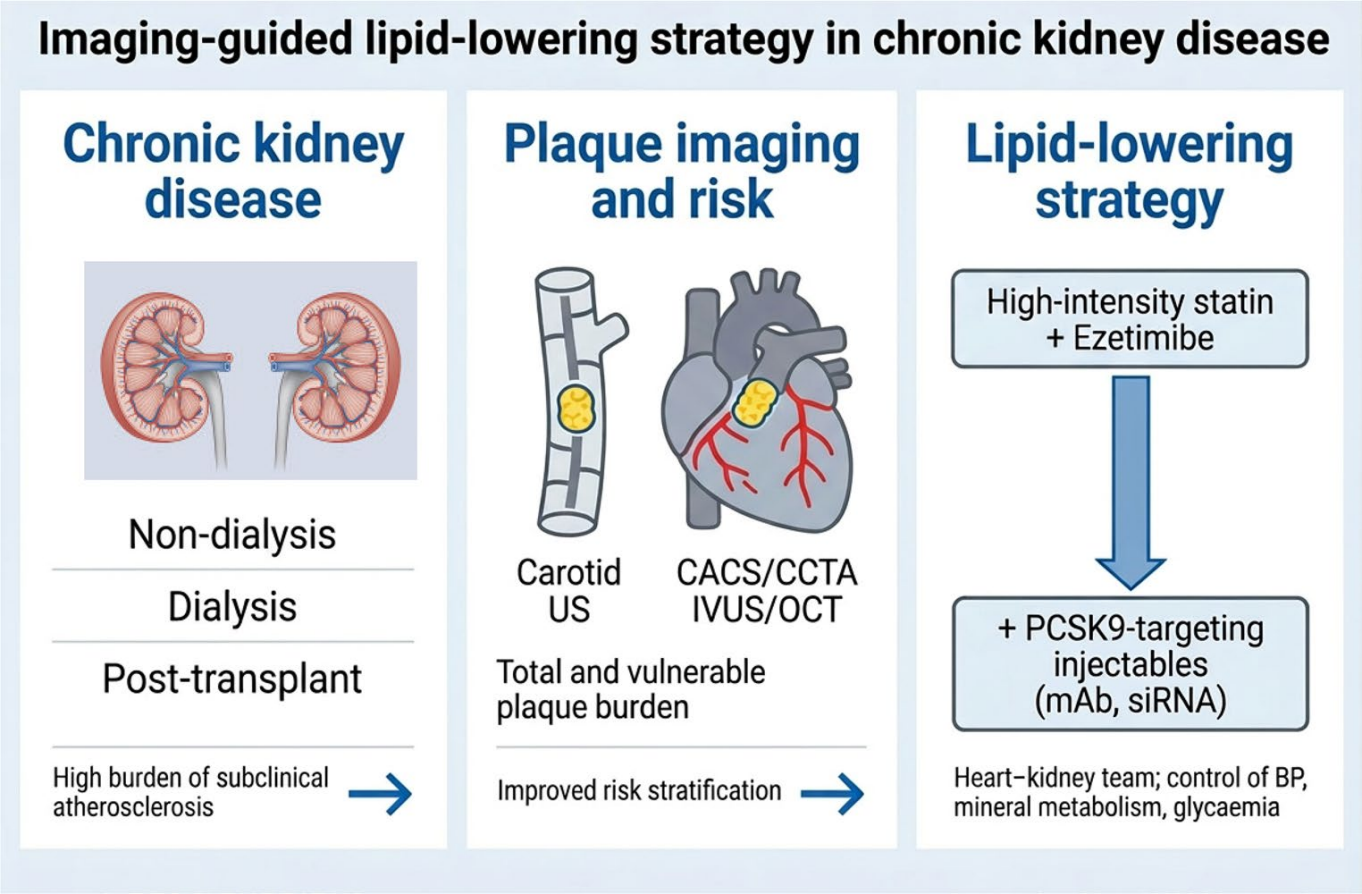
Lipid‑lowering strategy in chronic kidney disease. The schematic illustrates a three‑step pathway linking chronic kidney disease (CKD), atherosclerotic plaque imaging and treatment intensification. On the left, CKD (non‑dialysis, dialysis and post‑transplant) is associated with a high burden of subclinical atherosclerosis driven by dyslipidemia, inflammation and vascular calcification. In the central panel, carotid ultrasound, coronary artery calcium scoring and coronary CT angiography, supplemented by intravascular imaging (IVUS/OCT/NIRS) in selected patients, identify overall and vulnerable plaque burden, improving cardiovascular risk stratification beyond traditional clinical variables. On the right, the presence of extensive or high‑risk plaque informs a stepwise lipid‑lowering strategy: high‑intensity statins as the foundation, early addition of ezetimibe if LDL‑C targets are not achieved, and subsequent introduction of PCSK9‑targeting injectable therapies (monoclonal antibodies or small‑interfering RNA) to obtain deep and sustained LDL‑C reduction. The figure emphasizes that these decisions should be taken within a multidisciplinary heart–kidney care pathway, integrating imaging findings with comprehensive management of blood pressure, mineral metabolism and glycemia control

PCSK9 inhibitors and inclisiran offer long-acting LDL-C lowering with predictable adherence, and ongoing imaging studies are evaluating their impact on total atheroma volume and low-attenuation plaques [77]. In parallel, glucagon-like peptide-1 receptor agonists, originally developed as glucose-lowering agents, have demonstrated robust cardiovascular protection in high-risk patients, with emerging data suggesting favorable effects on body weight, blood pressure, inflammation, and possibly plaque biology [78, 79]. Within a multidisciplinary cardiorenal pathway, integrating coronary plaque imaging with these advanced injectable lipid- and risk-modifying therapies offers a concrete opportunity to individualize treatment intensity, systematically address residual risk, and potentially modify the natural history of atherosclerotic disease in patients with CKD (Fig. 2).

## Conclusions: The Patient Journey

In the context of CKD, the patient journey represents a dynamic, longitudinal care pathway in which cardiovascular risk progressively increases in parallel with declining renal function [80]. Dyslipidemia plays a major role, as it develops early in the course of CKD and more important represents a major, potentially modifiable determinant of the excess cardiovascular morbidity and mortality observed in this population [81, 82].

In line with current ESC/EAS recommendations, patients with CKD are categorized as being at high or very high cardiovascular risk, supporting the early initiation and long-term maintenance of lipid-lowering therapy, largely independent of baseline lipid levels [17]. However, the implementation of these recommendations across the CKD patient journey remains inconsistent. Competing clinical priorities related to renal disease management, concerns regarding drug safety and tolerability in the setting of impaired renal function, and therapeutic inertia frequently contribute to delayed initiation or suboptimal intensification of lipid-lowering treatment.

In patients with non-dialysis-dependent CKD, statin-based therapy, either alone or in combination with ezetimibe, represents the cornerstone of lipid management, with robust evidence supporting its role in cardiovascular risk reduction [33, 83].

As renal function declines, however, lipid abnormalities become increasingly complex, and key transitions in care -such as referral to nephrology services or progression to advanced CKD stages- constitute critical junctures at which lipid-lowering strategies should be systematically reassessed [83]. Despite clear recommendations, treatment start, maintenance and intensification as well as LDL-C target is frequently overlooked in CKD population [14, 84]. In patients initiating dialysis, the role of lipid-lowering therapy changes, as randomized clinical trials have demonstrated limited cardiovascular benefit from de novo statin initiation [51, 52]. Nevertheless, continuation of previously prescribed statin therapy is generally recommended, reflecting its established role earlier in the disease course [33] and the lack of evidence supporting routine discontinuation. Following kidney transplantation, dyslipidemia frequently worsens because of immunosuppressive regimens, thereby requiring renewed and individualized attention to lipid management [85]. In this setting, lipid-lowering strategies must carefully account for potential drug-drug interactions, particularly with calcineurin inhibitors and mTOR inhibitors, as well as for altered pharmacokinetics related to the improved but still fluctuating renal function.

A patient journey-based approach emphasizes the need for individualized, stage-specific lipid management that dynamically adapts to changes in renal function, comorbidities, and therapeutic objectives over time. Novel lipid-lowering therapies, including PCSK9 inhibitors, may provide additional therapeutic options for selected patients with CKD at very high cardiovascular risk, although real-world evidence in this population remains limited. Effective dyslipidemia management across the CKD patient journey relies on close multidisciplinary collaboration among cardiologists, nephrologists, and primary care physicians to ensure continuity of care and consistent implementation of evidence-based recommendations. Integrating lipid management into the broader, longitudinal care of patients with CKD is therefore essential to reduce cardiovascular morbidity and mortality, which remain the leading determinants of adverse outcomes across all stages of kidney disease.

## Key References

- Charytan DM, Sabatine MS, Pedersen TR, et al. Efficacy and Safety of Evolocumab in Chronic Kidney Disease in the FOURIER Trial. *J Am Coll Cardiol*. 2019;73(23):2961-2970.
  - ○ Provides evidence that evolocumab is effective and safe in patients with CKD.
- Tunnicliffe DJ, Palmer SC, Cashmore BA, et al. HMG CoA reductase inhibitors (statins) for people with chronic kidney disease not requiring dialysis. *Cochrane Database Syst Rev*. 2023;11(11):CD007784.
  - ○ Updated meta-analysis assessing benefits and harms of statins in patients with non-dialysis CKD.
- Ray KK, Reeskamp LF, Laufs U, et al. Combination lipid-lowering therapy as first-line strategy in very high-risk patients. Eur Heart J. 2022;43(8):830-833.
  - ○ Defines a treatment algorithm for very high-risk patients with ASCVD.
- Nicholls SJ, Kataoka Y, Nissen SE, et al. Effect of Evolocumab on Coronary Plaque Phenotype and Burden in Statin-Treated Patients Following Myocardial Infarction. *JACC Cardiovasc Imaging*. 2022;15(7):1308-1321.
  - ○ Examines the effect of evolocumab on coronary atherosclerotic plaque.

## Data Availability

No datasets were generated or analysed during the current study.
